# Internalizing problems are associated with oral health-related quality of life in early childhood: Outcomes from an Asian multi-ethnic prospective birth cohort

**DOI:** 10.1371/journal.pone.0256163

**Published:** 2021-08-12

**Authors:** Ruth Choe, Yu Fan Sim, Catherine H. L. Hong, Sameema Mohideen, Ranjani Nadarajan, Fabian Yap, Lynette P.-C. Shek, Chin-Ying Stephen Hsu, Birit F. P. Broekman, Joao N. Ferreira

**Affiliations:** 1 Faculty of Dentistry, National University of Singapore, Singapore, Singapore; 2 Singapore Institute for Clinical Sciences, A*STAR, Singapore, Singapore; 3 Department of Paediatrics, KK Women’s and Children’s Hospital, Singapore, Singapore; 4 Department of Paediatrics, Yong Loo Lin School of Medicine, National University of Singapore, Singapore, Singapore; 5 OLVG and Amsterdam UMC, VU University, Amsterdam, The Netherlands; 6 Faculty of Dentistry, Exocrine Gland Biology and Regeneration Research Group, Department of Research Affairs, Chulalongkorn University, Bangkok, Thailand; Virginia Commonwealth University, UNITED STATES

## Abstract

Oral health status ideally warrants for a holistic biopsychosocial approach to health and wellness. Little is known about the impact of behavioral problems on oral health-related quality of life (OHRQoL) in children due to the paucity of studies in early childhood, particularly in Asian multi-ethnic populations. This study evaluated the relationship between early child’s socioemotional factors and OHRQoL, as well as its association with orofacial pain (OFP) and early childhood caries (ECC) in the Asian GUSTO birth cohort. Mother-child dyads were postnatally assessed at 3 time points. The Child Behavior Checklist (CBCL) was used to assess the child’s socioemotional and behavioral problems at age 4–4.5 years together with other validated questionnaires to evaluate maternal anxiety and depression. ECC detection was performed at age 5, and OHRQoL (primary) and OFP (secondary) outcomes were assessed at age 6 from a total of 555 mother-child dyads. After a univariate regression analysis was performed to identify potential predictors and confounders, a multivariate regression model was run with predisposing factors (CBCL internalization and externalization problems, OFP, ECC) and adjusted for confounders (maternal psychosocial states, maternal education) to determine associations with OHRQoL. Results showed an association between CBCL internalization scores and poorer OHRQoL (RR = 1.03, p = 0.033, 95% CI 1.01 to 1.05), although the limited risk ratio may not have a practical applicability in psychosocially healthy children, alike the majority of those evaluated in this cohort. The average OHRQoL overall score among children with OFP was 2.39 times more than those without OFP (OR = 2.39, p < 0.001, 95% CI 2.00 to 2.86). Thus, in early childhood, OFP, and to lesser extent internalizing behaviors, may negatively impact OHRQoL. This study therefore highlights the complex relationship between OHRQoL and its predisposing socioemotional and somatic pain factors, and demands further investigations in clinically relevant populations.

## 1. Introduction

In the last decade, broad improvements in oral health have been reported in all World Health Organization (WHO) regions after the implementation of public policies focused on prevention at early life [1–3]. Despite this progress, early childhood caries (ECC) remains a prevalent childhood disease [4]. ECC commonly triggers orofacial pain complaints [5] resulting in chewing difficulties as well as a poor appetite [6] and diet [7] which subsequently impacts the quality of life of both the child and families [8]. Consequently, ECC and its associated orofacial pain and masticatory dysfunction pose a public health burden for oral care services [9–17]. To tackle these concerns within multi-ethnic Singaporean children, the Growing Up in Singapore Towards healthy Outcomes (GUSTO) cohort, a birth and mother-offspring longitudinal study, has comprehensively collected the oral health data and assessed the socioemotional well-being at early childhood [18,19].

For a more holistic reflection of patient and public healthcare needs, a biopsychosocial model for oral health is needed, one that goes beyond oral symptoms and diagnosis such as ECC. As such, the use of oral health-related quality of life (OHRQoL) instruments for the evaluation of oral health outcomes and treatment needs is common in craniofacial and dental research as well as in clinical practice as it holistically assesses subjective symptoms, functional and emotional well-being [20]. Children have to reach 8 years of age to fully understand and comprehend the tasks involved in reporting their health accurately within a 4-week recall period [21]; hence, to measure the OHRQoL before the age of 8, parents or guardians must be used as proxies, and assessment tools such as the Early Childhood Health Impact Scale (ECOHIS) can be utilized [22]. In addition to ECOHIS and OHRQoL dimensions, behavioral problems in children may also contribute to an impaired quality of life [23], with downstream implications on mental health later in life [24].

Child behavioral and socioemotional problems manifest as either internalizing problems, which include symptoms of anxiety/depression, somatization and emotional symptoms, or externalizing problems like attention-seeking problems or aggression [25]. Behavioral problems may considerably interfere with attitudes such as receptiveness to daily preventive measures e.g. toothbrushing or dietary modifications [26] which directly increases susceptibility to ECC, resulting in deterioration of oral health [27]. Child behavioral and socioemotional problems have also been shown to affect behaviors in the dental setting. In a recent cross-sectional study, authors found that children, aged 4 to 12 years old, with both internalizing and externalizing problems behaved more negatively during routine dental treatment [26]. Further, internalization problems have also been associated with higher instances of orofacial pains, abdominal pain [28–31], and other chronic pains elsewhere in the body [32–39]. Together with pain symptoms, internalization problems can predict for an impaired OHRQoL in 8- to 12-year old children [31]. However, the impact of pain somatization and internalizing problems on OHRQoL in pre-school children is not known due to the paucity of studies in early childhood. Thus, investigating the impact of child behaviors and internalizing problems on OHRQoL dimensions and understanding its relation to common pain reports (i.e. orofacial and abdominal) is of utmost importance. We hypothesize that child internalizing problems can have a negative impact on the child and parents OHRQoL.

Hence, this study aims (1) to identify whether child’s emotional and behavioral problems at age 4–4.5 years are associated with OHRQoL at age 6; (2) to determine if OHRQoL is associated with orofacial/abdominal pains at age 6; and (3) to investigate if ECC at age 5 can predispose for a poorer OHRQoL at age 6. This study report is based on the GUSTO cohort which is an ongoing mother-offspring cohort study that collects a comprehensive and wide array of phenotypic data including mental and oral health data from mothers and their offspring from pregnancy onwards [7,18,19].

## 2. Materials and methods

### 2.1 The GUSTO cohort and study design

The GUSTO is a multicenter longitudinal birth and mother-offspring cohort study based in the multi-ethic population of Singapore. It is one of the most comprehensive studies investigating the role of developmental and behavioral factors (including phenotypic, genetic and epigenetic factors) in health as well as oral health outcomes [7,18,19,40]. Deep phenotyping and longitudinal assessments of Singaporean mothers and their offspring was initiated in 2009 and is still ongoing, and such were evaluated from pregnancy onwards. Briefly, healthy pregnant women of Chinese, Malay or Indian and of homogeneous parental ethnic background were recruited during their first pregnancy trimester at the KK Women’s and Children’s Hospital (KKH) or National University Hospital (NUH) in Singapore from June 2009 to September 2010 (n = 1247). Ethics approval for the study was granted by the SingHealth Centralized Institutional Review Board (Reference No. 2009/280/D) for KKH and the National Health Care Group Domain Specific Review Board (Reference No. D/09/021) for NUH. Written informed consent was obtained from all women participants upon recruitment, and the study was conducted according to the principles of the Declaration of Helsinki.

### 2.2 Data collection

**Fig 1:**
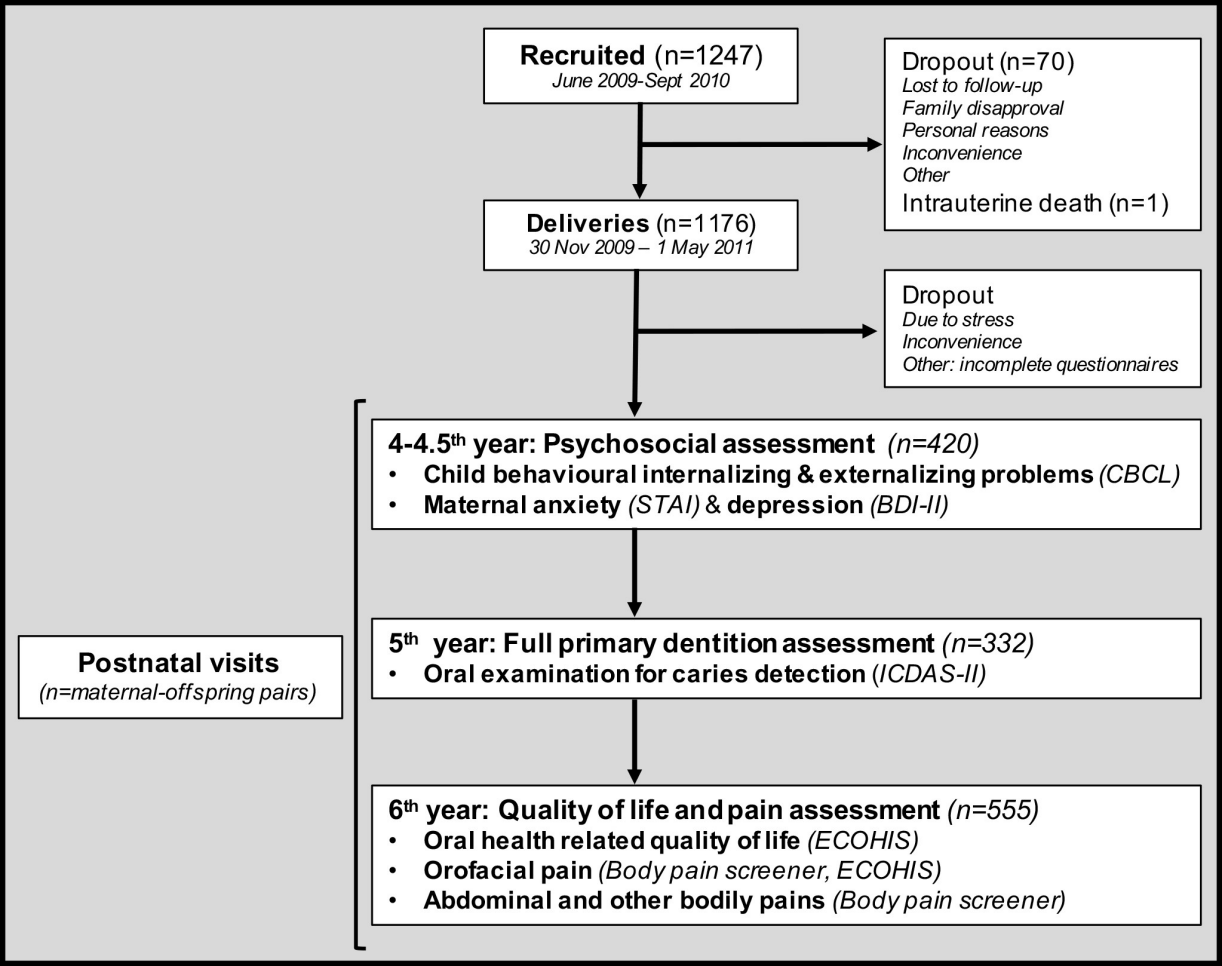
Flowchart with the longitudinal design and assessed variables at each postnatal visit of this study cohort.

In this cohort, mother-child dyads were postnatally assessed at 3 time points with oral examinations and on-site questionnaires at KKH and NUH as per study flowchart in Fig 1. All questionnaires were provided in Chinese, Malay, Tamil or English, after forward and backward translation to English by certified translation services.

### 2.3 Independent variables and predisposing factors

#### 2.3.1 Demographics

Demographic data such as ethnicity, gender and maternal education levels was collected at baseline, 4–4.5^th^- and 6^th^-year postnatal visits. Highest maternal education was coded as an ordinal variable according to the following categories/levels: below post-secondary, post-secondary and university level and above. These categories have been used in other published GUSTO studies [18,41–43] and served as an indicator of socio-economic status.

#### 2.3.2 Child behavioral and emotional problems

The Child Behavior Checklist 1.5–5 (CBCL/1.5–5) is a valid and reliable 99-item parent reported instrument used to screen for emotional and behavioral problems in 1.5- to 5-year old children [44,45]. In this study, responses were scored by the mothers on a 3-point Likert scale (0 = Not True, 1 = Somewhat or Sometimes True, 2 = Very True or Often True) at the 4–4.5^th^-year postnatal visit. A higher score means more symptoms of socioemotional and behavioral problems. This checklist evaluates the child’s internalizing (social withdrawal, emotionally reactive, somatic complaints and anxiety/depression scales) and externalizing problems (attention problems and aggressive behavior scales). Raw scores were converted to age-matched standardized T-scores using the Singapore-based norms [46]. The Cronbach’s alpha was 0.88 and 0.90 for the internalization and externalization problems subgroups respectively [47], indicating excellent internal reliability.

### 2.4 Covariates and potential confounders

#### 2.4.1 Maternal psychosocial problems

The self-reported clinical instrument Spielberger State-Trait Inventory (STAI) was used to detect maternal anxiety as previously [48] during the 4–4.5^th^-year postnatal visits. This instrument consists of 2 scales with 20 items; one scale appraises the current anxiety condition (State-Anxiety scale) while the other appraises the anxious personality traits (Trait-Anxiety scale). Each item of the STAI was scored on a 4-point Likert scale with higher scores signifying greater anxiety. The STAI has been shown to have construct validity [49] and reliability within the GUSTO cohort [50]. Internal consistencies were established within the cohort and the Cronbach’s alpha was 0.91 for both the State-Anxiety and Trait-Anxiety subscales [42].

The 21-item Beck Depression Inventory II (BDI-II) was used to assess the presence and severity of depressive symptoms in the preceding two weeks [51]. Each item of the BDI-II was scored on a 4-point Likert scale and summed as a total score (score range 0–63). Higher scores indicate more severe depressive symptoms. This widely used self-reported questionnaire has been validated to assess the existence and severity of depressive symptoms [51] and has been shown to have acceptable internal consistency in the literature (Cronbach’s alpha 0.73–0.95) [52].

A higher score on both the STAI and BDI-II means more maternal symptoms of anxiety and depression are present.

#### 2.4.2 Early childhood caries

During the 5^th^-year postnatal visits, oral examinations were performed by 3 calibrated dentists (intraclass correlation coefficient (ICC): >0.80) using the modified International Caries Detection and Assessment System (ICDAS-II). Early childhood caries (ECC) was assessed by the number of decayed teeth with incipient caries or cavitated lesions corresponding to ICDAS-II codes 2–6 [53]. Children refrained from consuming any foods, drinks or toothbrushing for at least 1 hour before the oral examination. Participants were examined in a supine position on the dental chair. Teeth surfaces were cleaned, dried with sterile gauze and assessed by visual examination using mouth mirrors and torchlights for artificial illumination. Tactile inspection with WHO blunt probes were used to aid the visual examination when necessary. No radiographs were taken.

#### 2.4.3. Orofacial pain and other bodily pains

At the 6^th^-year postnatal visit, the presence and frequency of orofacial pain in children was assessed by the question: “How often has your child had pain in the teeth, mouth or jaws?”. The use of this question to measure pain has been widely used in epidemiological orofacial pains studies [54–56] and has been validated as a pain construct in ECC and OHRQoL studies and tools [57]. The presence of parental-reported orofacial pain was derived from the question and dichotomized. Those who answered “never” or “hardly ever” were categorized as children with “no orofacial pain” while the rest of the responses were recorded as having “orofacial pain”.

An interviewer-administered body pain drawing and screener was then used to directly assess the child’s orofacial pain, abdominal pain and other bodily pains in specific anatomical locations within the last month. This screening tool was adopted from the International Network for Orofacial Pain and Related Disorders Methodology and the International Classification of Orofacial Pain [58,59].

### 2.5 Outcome variables

#### 2.5.1 Oral health-related quality of life

The ECOHIS was administered to mothers at the 6^th^-year postnatal visit. Mothers were asked to consider the child’s entire lifespan when answering the questionnaire. The 13-item questionnaire consists of 2 sections: the child (9 items) and family (4 items) impact sections. Response categories were scored on a 5-point Likert scale: 0 = never; 1 = hardly ever; 2 = occasionally; 3 = often; 4 = very often; 5 = don’t know; “don’t know” responses were recorded as missing and excluded from the overall ECOHIS score. The overall ECOHIS score is calculated based on the summation of the response codes for the family and child sections; whereby higher scores indicate poorer OHRQoL. Subjects with more than 2 missing responses in the child section or 1 missing in the family section were excluded [22]. Crohnbach alphas for child and family sections were 0.91 and 0.95 respectively and the ICC was 0.84 [22].

### 2.6 Data analysis

STATA SE Version 15 (StataCorp LLC, College Station, Texas, USA) was utilized for all analyses and the significance level was set at *p* < 0.05.

The primary outcome measure, OHRQoL, was analyzed as a quantitative variable, and the scores on CBCL, STAI and BDI-II as well. ECC was measured as a quantitative variable by summation of the number of decayed teeth (dt) with white spots/cavitated lesions (ICDAS-II codes 2–6). We assessed the correlation between CBCL and OHRQoL and performed univariate regression analyses to investigate the relationship between the primary outcome measure, OHRQoL, and independent variables (orofacial pain, abdominal pain and ECC).

Lastly, a multivariate regression model was run with potential predisposing factors or predictors (CBCL internalization and externalization problems) and adjusted for confounders arising from the univariate analysis (e.g., independent variables with a significant association with the primary outcome measure, OHRQoL, exhibiting a p-value < 0.05). As the OHRQoL, a discrete variable that takes only non-negative values, had positively skewed distribution with overdispersion, generalized linear model with negative binomial family distribution was considered. Negative binomial regression analysis with log-linked function and robust variance estimator was used to estimate the association, in terms of risk ratio (RR) with 95% confidence interval (CI), between independent variables and OHRQoL. Negative binomial regression analysis was deemed suitable for the data set examined and fulfilled the assumptions in the analysis.

To account for potential bias from missing data and for comparable purposes with our previous cohort published studies, multiple imputation using chained equations was employed under the assumption that data were missing at random conditional on the observed data. Forty imputed datasets were generated from imputation models containing all potential predisposing factors and confounders included in the regression analysis. Number of imputations were determined at Monte Carlo error was <10% of the standard error of the estimates to achieved convergence of the parameter estimates. Predictive mean matching algorithms, which is robust against misspecification of the imputation model, was used for imputation of values. Regression analysis were conducted on imputed datasets on final sample of n = 555 and estimates were combined following Rubin’s rule into a single estimate which is less biased by differential losses to follow up. In addition to this protocol, a planned sensitivity analyses using complete cases was performed to assess the robustness of the findings from aforesaid regression models.

## 3. Results

### 3.1 Sociodemographics

A total of 555 mothers completed all questionnaires in this cohort at the last postnatal visit. Majority of mothers had higher education levels with 38.9% being university degree holders. The children had a male-to-female ratio of 1:1.2 and were predominantly Chinese (53.2%).

The mean OHRQoL scores was 5.51 (Median: 3.00, range: 0–32) (S1 Table). The lifetime prevalence of orofacial pain and abdominal pain in the birth cohort was 23.1%, and 43% respectively (S1 Fig). The mean CBCL total score was 50.62 (SD: ±11.02, range: 0–125). The mean maternal STAI and BDI-II were 70.42 (SD: ±19.42) and 6.60 (SD: ±7.79) respectively (S2 Table).

Sociodemographic characteristics of the primary outcome OHRQoL are presented in Table 1. Maternal education was associated with overall OHRQoL (p = 0.017) and OHRQoL family subscale (p = 0.008), hence maternal education may have a protective role.

**Table 1:** Sociodemographics and their association with OHRQoL. OHRQoL overall was calculated from overall sum of ECOHIS scores.

Sociodemographic variables	OHRQoL					
	Overall		Child		Family	
	*Mean (SD)*	*p-value*	*Mean (SD)*	*p-value*	*Mean (SD)*	*p-value*
***Child’s Gender***						
*Male*	5.27 (5.88)	0.468	4.06 (4.35)	0.609	1.22 (2.05)	0.229
*Female*	5.79 (6.24)		4.28 (4.43)		1.51 (2.38)	
***Child’s Ethnicity***						
*Chinese*	5.39 (5.55)	0.740	4.00 (4.03)	0.997	1.42 (2.04)	0.081
*Malay*	5.82 (6.88)		4.40 (4.82)		1.42 (2.61)	
*Indian*	5.32 (6.02)		4.28 (4.65)		1.04 (1.94)	
***Maternal Education***						
*< Post-secondary*	6.30 (6.84)	0.017**	4.62 (4.86)	0.076	1.63 (2.52)	0.008**
*Post-secondary*	4.21 (4.93)		3.34 (3.73)		0.85 (1.64)	
*≥ University*	5.36 (5.03)		3.94 (3.72)		1.42 (1.95)	

Thirteen parent-child dyads had missing data on ethnicity and gender, and 119 responses had missing data for maternal education. The CBCL was incomplete in 135 responses. No cases were excluded, and incomplete cases were accounted for in the multiple imputation model.

### 3.2 OHRQoL relationships with pain and ECC

Relationships between OHRQoL and bodily pains (orofacial and abdominal) and ECC were assessed (Table 2). Children with orofacial pain (OFP) were associated with higher OHRQoL (overall) as well as with child and family impact scores (p < 0.001). Children with ECC were also correlated with higher OHRQoL (p = 0.006) but only had negative impact on the child scores (p = 0.004).

**Table 2:** Mann-Whitney U test to identify the relationships between orofacial pain, abdominal pain, ECC and OHRQoL (overall and subscales).

*Pain-related Variables*	OHRQoL					
	Overall		Child		Family	
	*Mean (SD)*	*p-value*	*Mean (SD)*	*p-value*	*Mean (SD)*	*p-value*
**Orofacial Pain**		<0.001**		<0.001**		<0.001**
Yes	10.63 (7.28)		8.12 (5.14)		2.51 (2.93)	
No	3.97 (4.62)		2.95 (3.31)		1.02 (1.83)	
**Abdominal Pain**						
Yes	6.04 (6.04)	0.125	4.53 (4.68)	0.285	1.51 (2.06)	0.224
No	5.43 (5.99)		4.09 (4.31)		1.34 (2.24)	
**ECC (ICDAS-II)**						
Yes	5.79 (6.27)	0.006**	4.44 (4.37)	0.004**	1.35 (2.38)	0.278
No	4.17 (5.13)		3.21 (3.73)		0.96 (1.78)	

Abbreviations: ECC, Early Childhood Caries; ICDAS-II, International Caries Detection and Assessment System-II.

### 3.3 Regression analysis

With multiple imputation method for missing data, a univariate regression analysis was performed to identify potential predictors and confounders (Table 3). CBCL internalizing, externalizing and total scores were associated with a higher OHRQoL overall score. However, an increase of 1-unit score in the CBCL total score was only linked with an average 2% increase in the OHRQoL overall score and the 95% CI were very narrow (RR = 1.02, *p* < 0.001, 95% CI 1.01–1.03). This relationship with limited RR was also observed with both the OHRQoL impact scores on the child *(*RR = 1.01, p < 0.001, 95% CI 1.01–1.02) and family (RR = 1.01, p < 0.001, 95% CI 1.00–1.02) (S3 Table).

**Table 3:** Univariate regression analysis for variables associated with overall OHRQoL.

*Potential Predisposing Factors/Confounders*	OHRQoL		
	*RR* ^*U*^	*95% CI*	*p-value*
**CBCL Internalization**	1.03	1.02 to 1.05	<0.001**
Somatization	1.07	1.02 to 1.11	0.003**
Withdrawn	1.10	1.06 to 1.14	<0.001**
Emotional	1.11	1.06 to 1.15	<0.001**
Anxiety & Depression	1.10	1.05 to 1.14	<0.001**
**CBCL Externalization**	1.03	1.01 to 1.04	<0.001**
Aggressive	1.03	1.01 to 1.13	<0.001**
Attention	1.07	1.01 to 1.13	0.025**
**CBCL Total score**	1.02	1.01 to 1.03	<0.001**
**Maternal STAI**	1.01	1.00 to 1.02	0.001**
**Maternal BDI-II**	0.99	0.98 to 1.01	0.482
**Maternal education**			
*< Post-secondary*	1		
*Post-secondary*	0.66	0.50 to 0.87	0.003**
*≥ University*	0.87	0.67 to 1.12	0.268
**Orofacial pain**	2.67	2.27 to 3.14	<0.001**
**Abdominal pain**	1.11	0.87 to 1.43	0.403
**ECC (ICDAS-II)**			
*No*	1		
*Yes*	1.41	1.10 to 1.79	0.006**

^U^Unadjusted risk ratio from univariate regression analysis by negative binomial regression model.

(RR: Risk Ratio, CI: Confidence Intervals). Abbreviations: CBCL, Child Behavioral Checklist; STAI, State-Trait Anxiety Inventory; BDI-II, Beck Depression Inventory Second Edition; ECC, Early Childhood Caries; ICDAS-II, International Caries Detection and Assessment System-II.

The average OHRQoL overall score in children with OFP was 2.67 times higher than those without OFP (RR = 2.67, *p* < 0.001, 95% CI 2.27 to 3.14) (Table 3). Moreover, the average OHRQoL overall score in children with ECC was 1.41 times higher than those without ECC (p = 0.006) (Table 3).

In the multivariate regression model (Table 4), predisposing factors (CBCL internalization and externalization) and outcome variables (OHRQoL) were included while adjusting for multiple factors (maternal STAI and education, OFP, ECC), all according to the univariate analysis findings. CBCL total score was removed due to multicollinearity. CBCL internalization scores remained associated with poorer OHRQoL (RR = 1.03, p = 0.033, 95% CI 1.01 to 1.05), but not externalization. CBCL internalization score was also correlated with the ECOHIS child subscale (RR = 1.03, p = 0.002, 95% CI 1.00 to 1.05) and the family subscale (RR = 1.03, p = 0.030, 95% CI 1.00 to 1.06). Though, RR and 95% CI were near 1 for all the above relationships of CBCL with OHRQoL. Moreover, the average ECOHIS overall score in children with OFP was 2.39 times higher than those without OFP (OR 2.39, p < 0.001, 95% CI, 2.00–2.86).

**Table 4:** Multivariate regression analysis for all relevant variables associated with OHRQoL dimensions by multiple imputation modeling.

*Variables*	OHRQoL								
	*Overall*			*Child*			*Family*		
	*RR* ^*A*^	*95% CI*	*p-value*	*RR* ^*A*^	*95% CI*	*p-value*	*RR* ^*A*^	*95% CI*	*p-value*
**CBCL Internalization**	1.03	1.01 to 1.05	0.033**	1.03	1.00 to 1.05	0.002**	1.03	1.00 to 1.06	0.030**
**CBCL Externalization**	0.99	0.97 to 1.01	0.205	0.99	0.97 to 1.01	0.270	0.98	0.95 to 1.01	0.259
**Maternal STAI**	1.01	1.00 to 1.01	0.135	1.01	1.00 to 1.01	0.105	1.00	0.99 to 1.01	0.469
**Maternal education**									
*< Post-secondary*	1			1			1		
*Post-secondary*	0.84	0.64 to 0.10	0.197	0.91	0.70 to 1.18	0.489	0.62	0.39 to 0.97	0.038**
*≥ University*	0.97	0.67 to 1.42	0.513	1.04	0.82 to 1.32	0.753	1.19	0.81 to 1.74	0.384
**Orofacial pain**	2.39	2.00 to 2.86	<0.001**	2.46	2.08 to 2.91	<0.001**	2.22	1.63 to 3.04	<0.001**
**ECC**	1.26	0.98 to 1.62	0.066	1.29	1.00 to 1.66	0.046**	1.20	0.83 to 1.76	0.332

^A^: Adjusted risk ratio from multivariate regression analysis by negative binomial regression model.

CBCL internalization and externalization were input as predisposing factors for OHRQoL outcomes, which were adjusted for maternal STAI, maternal education, Orofacial pain and ECC variables. Abbreviations: CBCL, Child Behavioral Checklist; STAI, State-Trait Anxiety Inventory; ECC, Early Childhood Caries.

In addition to the multiple imputation analysis, a sensitivity analysis using complete cases was performed as well to confirm the regression models (S4 Table). We found that results from both multiple imputation modelling (Table 4) and sensitivity analysis (S4 Table) were comparable for the main hypotheses that internalizing behaviors, but not externalizing, are associated with poorer OHRQoL. Despite these findings, the RR is near 1 for both the imputation model and sensitivity analysis. Moreover, OHRQoL is also associated with OFP, independent of ECC and other bodily pains. ECC approached significance in its relationship with OHRQoL, however the 95% CI are very wide in both analyses (Tables 4 and S4).

## 4. Discussion

The role of behavioral and emotional problems on OHRQoL in children is unclear due to the dearth of early childhood studies in dentistry. To our knowledge, this was the first study to examine the effect of the child’s socioemotional and behavioral problems (measured by CBCL) on OHRQoL in the context of bodily pains and early childhood caries (ECC). Herein, we found that higher internalizing CBCL scores in 4-year-old (±5 months) children were negatively associated with the OHRQoL, which in turn (poorer OHRQoL) was associated in children with orofacial pain. From the limited data extrapolated from pediatric temporomandibular disorders (TMD) [60,61], children with symptomatic TMD conditions had worse behavioral problems than those with asymptomatic and non-painful TMD conditions [60–62]. This is also reported in pain studies from the medical literature, whereby internalization problems, in particular, are associated with higher instances of comorbidities such as recurrent abdominal pain (RAP) [63], headaches, musculoskeletal pain and juvenile rheumatoid arthritis [64]. The hypothesis for this observation is that children with internalization phenotypes are often hypervigilant and tend to ruminate about their pain giving rise to greater pain sensitivity and amplified pain responses. This theory was demonstrated in a study comparing children with low-level versus high-level dental anxiety [65]. Children with a higher baseline level of dental anxiety reported more negative thoughts about pain during a dental restoration which reinforced the pain-related rumination [25,65]. In this cohort study, there was an association between CBCL internalization scores and poorer OHRQoL, although an increase of 1-unit score in the CBCL internalization score was only linked with a 3% increase in the ECOHIS overall score. These findings are consistent with literature as, though limited, there is a general observation that socioeconomic and behavioral problems, internalization problems in particular, may hinder the child’s coping mechanism and influence their behaviors and responses (e.g., intensification) to future painful experiences [25,66–72]. As such, dysregulation of pain modulation pathways caused by anxiety or mood disorders could possibly underlie the hypothesized association between internalization problems and development of chronic pain and comorbid conditions [73]. This may therefore serve as a possible explanation for the observed association between only internalization factors, not externalization, and quality of life. Another possible explanation for the lack of association between externalization with quality of life might be because externalization problems in children are hypothesized to be outcomes of an accumulation of predictors such as recurrent parental distress (maternal and paternal depression) [74], child emotional reactivity [75] and family dysfunction [76]. A child’s social and coping skills are heavily influenced by parents through observational learning and modelling [77]. Negative parental responses to pain or stress may unknowingly reinforce maladaptive coping strategies and lead to poorer social competence and adjustment issues in adolescence [74]. While studies in the literature have shown that there is no gold standard for assessment of childhood psychosocial disorders [76], future studies could consider assessment of parental distress and family dysfunction for a more holistic assessment of externalization issues in children and its effect on overall quality of life.

Although a positive correlation between the increase in CBCL internalization scores and poorer OHRQoL was established, it would be overly simplistic to directly extrapolate this data to clinically meaningful data. Established CBCL cut-offs for identifying children with normal/non-clinical (CBCL total scores <60), borderline (CBCL total scores = 60–63) or clinical (CBCL total scores >63) have been reported in clinical studies [44,46]. Our cohort comprised largely of a mentally healthy population with limited pain chronicity, absence of borderline maternal psychological conditions and a relatively restricted number of children falling below the borderline or high scores above the clinical cut-offs, therefore dichotomizing CBCL may result in skewed data analysis and lack of power (mean CBCL total score = 50.63, SD: ±11.02). Nonetheless, our study established that an increase in internalizing behaviors, even within the normal range, are associated with OHRQoL in early childhood, which is relevant to the population at large.

Various instruments are available to evaluate oral health problems in young adult and geriatric populations [78]. As children are constantly in transitional phases of emotional, cognitive, and social development, several considerations arise when reporting OHRQoL in children. In children, the assessment of quality of life is more complex as firstly, the comprehension of the questionnaire is dependent on the proxy’s age and cognitive development [79]. Secondly, the perceptions, expectations and emotional states of parents or caregivers have to be taken into account which may affect the accuracy of the ECOHIS questionnaire. Currently, the ECOHIS [22] is the only validated instrument to measure OHRQoL in children below 8 years of age that require parents or guardians as proxies due to their poor perception of health [21]. While the ECOHIS has been used widely in epidemiological studies, the instrument does not fully account for certain inherent behavioral problems which may confound the child’s reporting of pain and perceptions [80,81]. Out of the 13-items assessed by ECOHIS, only one assesses for the pain dimension [22]. Pain perceptions are shaped by the amalgamation of learned occurrences, memories of past experiences and pain coping approaches as the child grows up and develops neurocognitive skills [82,83]. Child behaviors may vary during their complex neurodevelopmental stages and hence may have an effect on their orofacial pain awareness and report [84,85]. This highlights the complexity and multidimensional experience of psychosocial problems on OHRQoL and orofacial pain which is unique to the individual patient. Hence, an understanding of how internalizing problems may predict the OHRQoL and associations with pain reports provide a more holistic and comprehensive understanding of the psychosocial effects on OHRQoL and pain pathways.

ECC in the primary dentition was found to be associated with poorer OHRQoL but such relationship did not hold in the multivariate regression model. Although the relationship between caries experience and a corresponding decrease in OHRQoL is well documented in the literature [9,12–17], only a few studies have explored the impact of odontogenic pain on OHRQoL in children [8,10,86]. A cross-sectional Brazilian study reported that parents of children with a history of dental pain had an 84-fold chance of reporting a negative impact on the child’s OHRQoL [10]. The study also demonstrated that a history of dental pain was a stronger predictor of OHRQoL than caries. Hence, an evaluation of both objective (e.g. caries) and subjective measures (e.g. pain symptoms) and their interactions is a more holistic management approach to understand the impact of orofacial pain predictors on overall OHRQoL. In this study, ECC was measured using the ICDAS-II which differentiates between enamel (ICDAS 1–3) and dentine lesions (ICDAS ≥ 4) [87]. This indicator is an accurate reflection of caries severity resulting in pain episodes, which arise when carious lesions progress from dentine to pulpal tissue [88,89]. However, and according to previous GUSTO publications, ECC measured with the ICDAS-II index was dichotomized into binary variables: absent (ICDAS 0) or present (ICDAS 2–6) [7]. Thus, it was not possible to retrospectively dichotomize subjects with superficial and deep lesions. Consequently, this may have led to a less clear relationship between ECC and OHRQoL (wide 95% CI), though such relation did approach a significance level (p = 0.066). Moreover, the ICDAS-II does not capture premature tooth loss due to extraction of carious teeth due to infection or dental trauma in the total score which may also be a contributory cause for orofacial pain and poorer OHRQoL [90,91]. Though, dental trauma at 6–7 years of age has low frequency rates in Singapore [92] and this cohort’s oral examinations did not depict any gross dental trauma case. Despite these limitations with ICDAS-II, this index is still the most widely used tool for reporting the dental disease burden in cohort studies [93]. Future studies should consider including clinical variables such as tooth loss due to premature extraction which may possibly contribute to OHRQoL and OFP [8,94].

In large population-based studies, abdominal pain is one of the most common pain complaints during childhood and it is typically associated with other somatic pain symptoms and internalizing disorders (i.e. child and maternal anxiety) [95]. These abdominal pains can be exacerbated by diet, including milk and carbohydrate intolerances [96], and previous GUSTO studies [7] have shown that such dietary patterns are linked with an increase in EEC which detrimental for an optimal oral health. Though, in this cohort, abdominal pain was not associated with OHRQoL at age 6. Further studies should focus on clinical cohorts of pre-school children with high prevalence of persistent or chronic pains to provide a more robust explanation for the relationship between behavioral and emotional problems and OHRQoL and its association with common pain complaints in the orofacial and abdominal regions. A strength of this study is that our findings were based on a longitudinal birth cohort and thus are likely to be representative for children and families in multi-ethnic communities. This study was also able to capture sociodemographic information (gender, ethnicity and socioeconomic status) that are important for the assessment and prediction of psychosocial factors on OHRQoL and its association with orofacial pain. In fact, herein maternal education was indeed a protective factor for OHRQoL, which fits in reported multilevel conceptual models on children’s oral health [97,98]. However, further studies are needed to establish whether the relationship between the child’s psychosocial factors and OHRQoL may be more robust in clinical subjects with chronic orofacial pain and/or comorbid psychosocial conditions such as psychopathology. These may provide more information on potential risk factors on the genesis of pain episodes later in life. Identification of these psychosocial variables will allow the clinicians together with parents to better understand and formulate interventions to help the child develop healthy pain coping behaviors aimed at improving the overall quality of life. Interventions may include the modification of anxious expectations or dysfunctional patterns of responses while facing pain symptoms. The understanding of how internalizing problems may predict the OHRQoL and associations with pain reports provide a more holistic and comprehensive understanding of the psychosocial effects on OHRQoL and pain pathways which may, in turn, prevent downstream implications on mental health later in life.

## 5. Conclusion

This cohort study identified that internalizing behaviors (i.e., somatization, withdrawn, emotional, anxiety and depression), but not externalizing behaviors in early childhood were associated with poor OHRQoL, where a poorer OHRQoL was associated with children with orofacial pain, independent of caries and common pain complaints from the abdominal regions. As the child’s internalizing problems may vary during different neurodevelopmental stages, this may have an effect on their orofacial pain awareness and reporting. This therefore highlights the complexity and multidimensional experience of internalizing problems on OHRQoL and orofacial pain which is unique to the individual patient.

## Supporting information

S1 Fig Frequency of different self-reported pains outside the orofacial region within the last month in Singaporean children at the 6^th^ year visit (n = 555). (TIF)

S1 Table Overall and domain specific ECOHIS scores. (TIF)

S2 Table Child emotional and behavioral problems and maternal psychosocial problems at the completion of primary dentition years. (TIF)

S3 Table Univariate regression analysis for variables associated with OHRQoL child and family subdomains. Result by negative binomial regression model. ^U^Unadjusted risk ratio from univariate regression analysis by negative binomial regression model. Abbreviations: CBCL, Child Behavioral Checklist; STAI, State-Trait Anxiety Inventory; BDI-II, Beck Depression Inventory Second Edition; ECC, Early Childhood Caries; ICDAS-II, International Caries Detection and Assessment System-II.
(TIF)

S4 Table Multivariate regression analysis for all relevant variables associated with OHRQoL dimensions using a sensitivity analysis with complete cases. (TIF)
