# Correction: Internalizing problems are associated with oral health-related quality of life in early childhood: Outcomes from an Asian multi-ethnic prospective birth cohort

**DOI:** 10.1371/journal.pone.0311236

**Published:** 2024-09-24

**Authors:** Ruth Choe, Yu Fan Sim, Catherine H. L. Hong, Sameema Mohideen, Ranjani Nadarajan, Fabian Yap, Lynette P.-C. Shek, Chin-Ying Stephen Hsu, Birit F. P. Broekman, Joao N. Ferreira

There is an error in affiliation 6 for author Joao N. Ferreira. The correct affiliation 6 is: Exocrine Gland Biology and Regeneration Research Group, Department of Research Affairs, Faculty of Dentistry, Chulalongkorn University, Bangkok, Thailand.

The following information is missing from the Funding statement: C.-Y. S. Hsu was supported by the National University Health System under its NUHS Bridging Funds 02/FY16 by grant number NUHSRO/2017/022/Bridging/04: R-221-000-110-733, and Singapore’s National Medical Research Council by grant number NMRC/CIRG/1341/2012: R-221-000-059-511.
