# Supplementary figures and images for: Internalizing problems are associated with oral health-related quality of life in early childhood: Outcomes from an Asian multi-ethnic prospective birth cohort
Source: PLoS One. 2021 Aug 12;16(8):e0256163. doi: 10.1371/journal.pone.0256163 (PMC8360536; doi:10.1371/journal.pone.0256163)

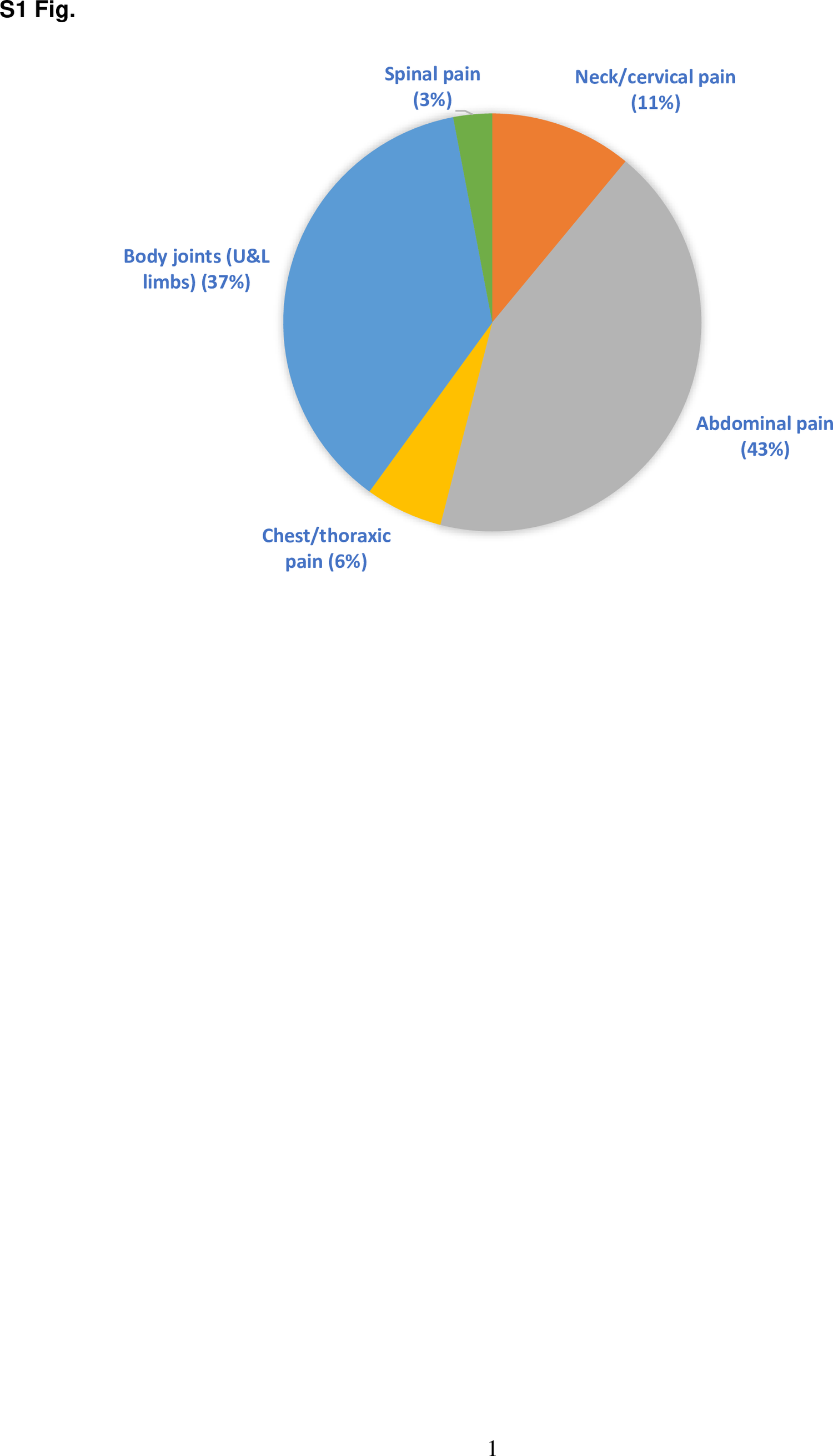

Supplement: S1 Fig — (TIF) [file pone.0256163.s001.tif]

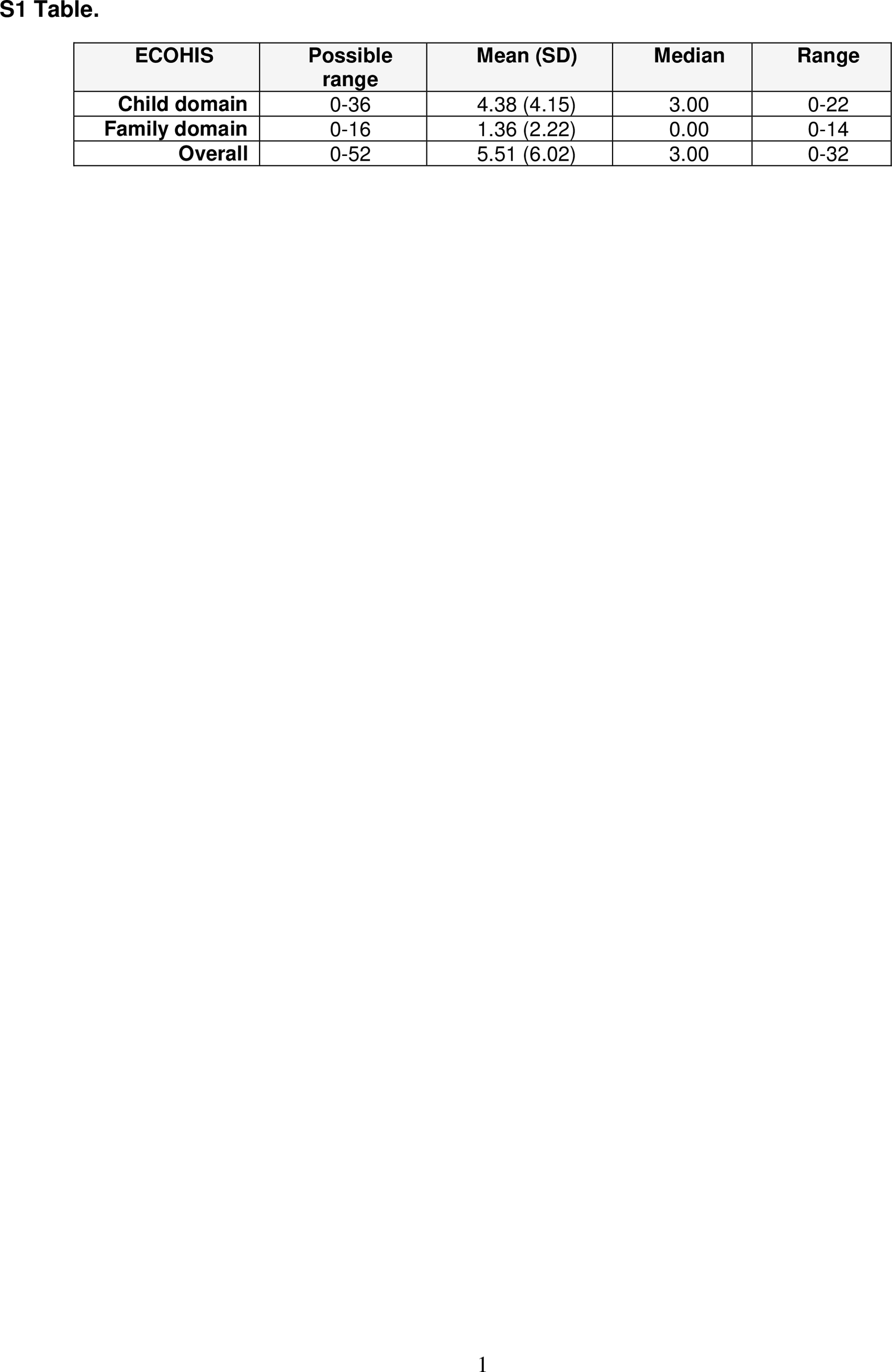

Supplement: S1 Table — (TIF) [file pone.0256163.s002.tif]

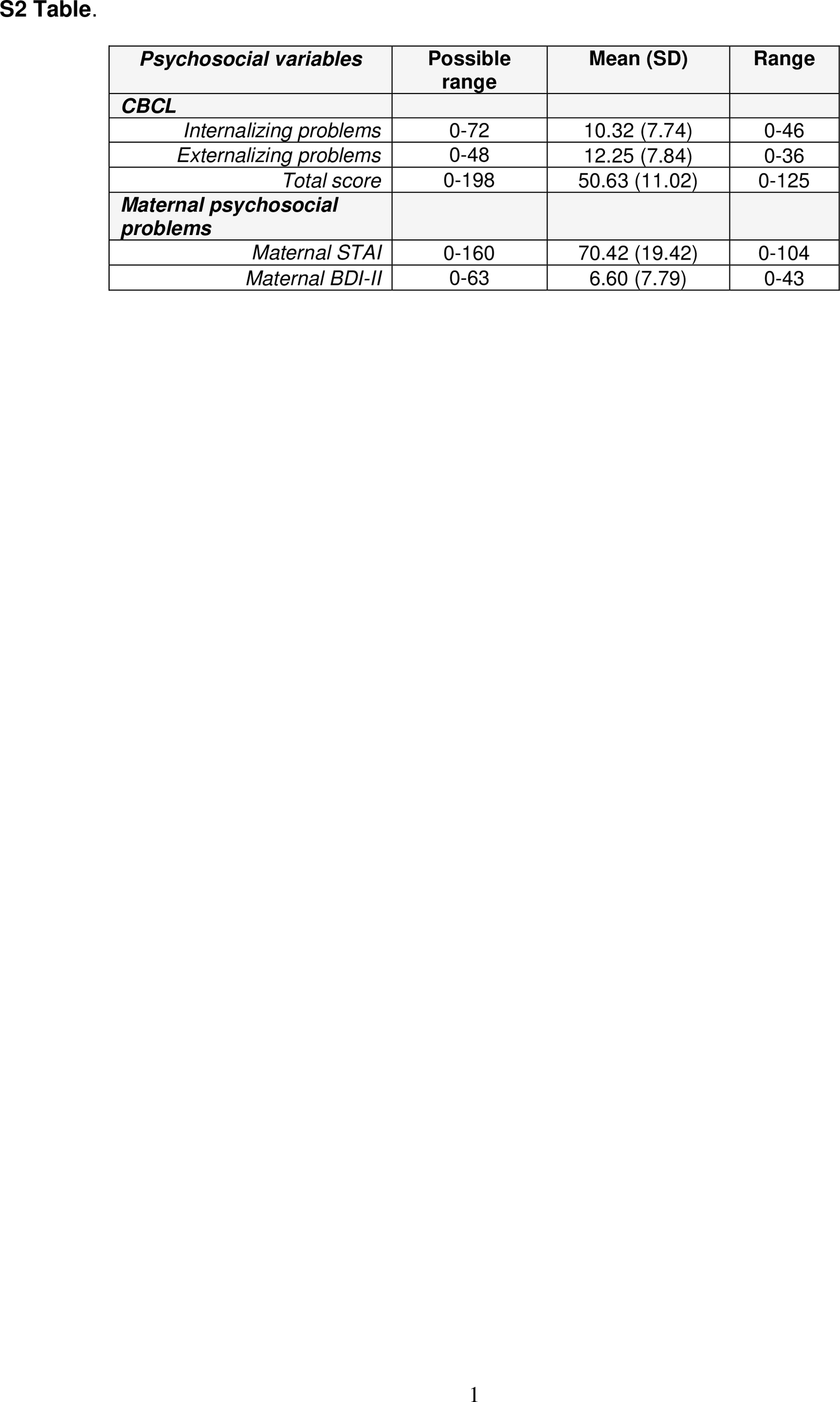

Supplement: S2 Table — (TIF) [file pone.0256163.s003.tif]

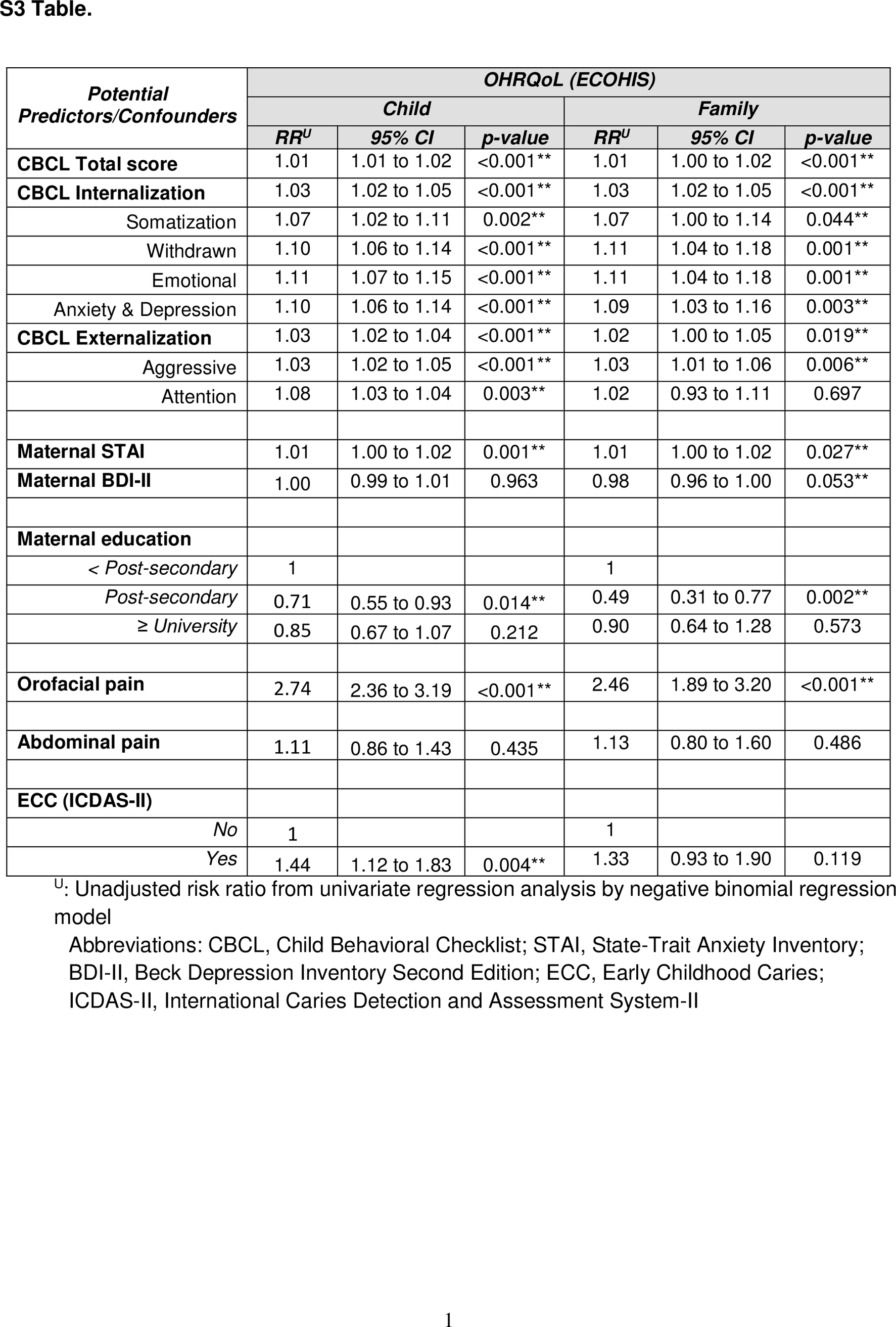

Supplement: S3 Table — Result by negative binomial regression model. UUnadjusted risk ratio from univariate regression analysis by negative binomial regression model. Abbreviations: CBCL, Child Behavioral Checklist; STAI, State-Trait Anxiety Inventory; BDI-II, Beck Depression Inventory Second Edition; ECC, Early Childhood Caries; ICDAS-II, International Caries Detection and Assessment System-II. (TIF) [file pone.0256163.s004.tif]

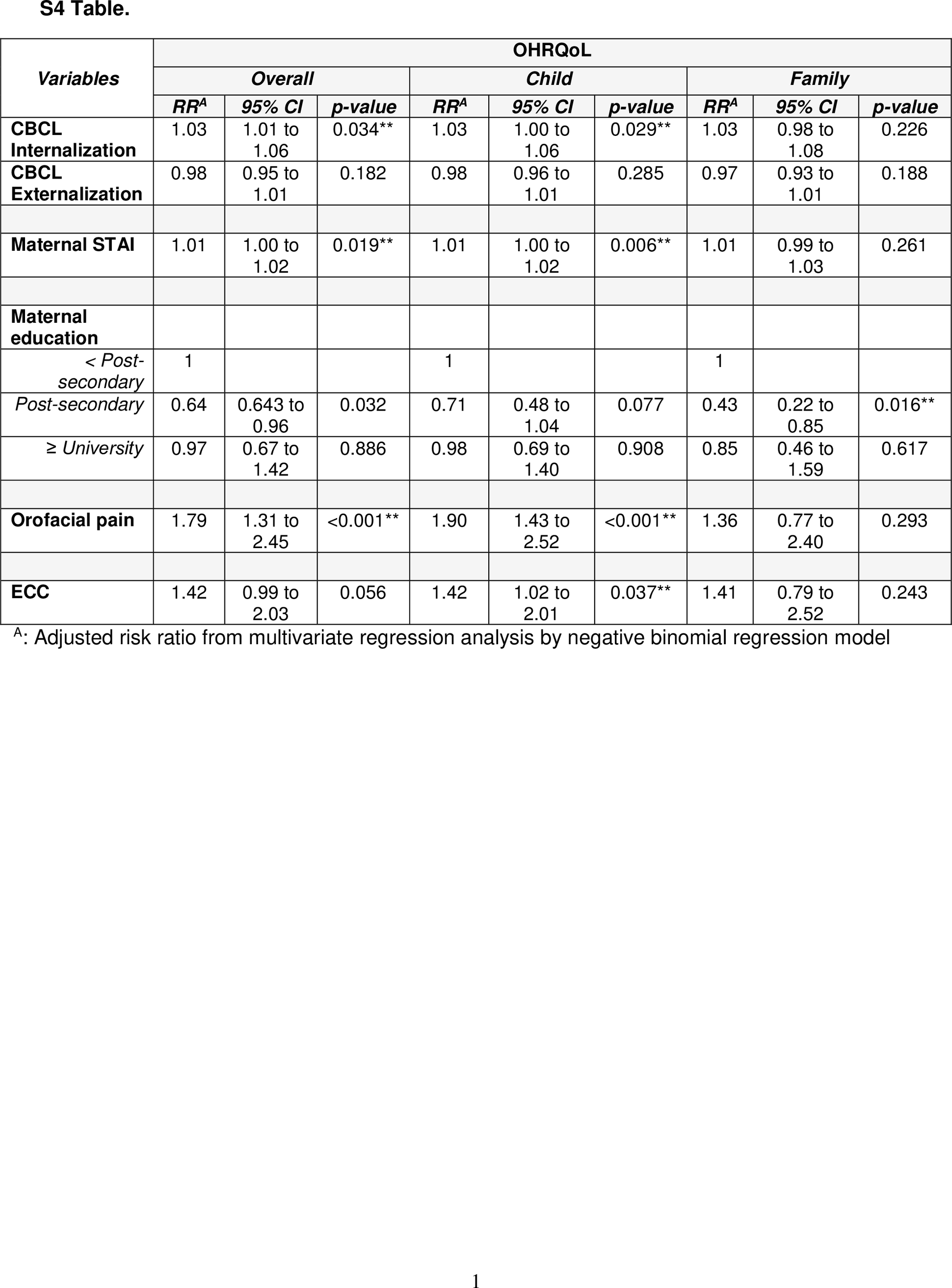

Supplement: S4 Table — (TIF) [file pone.0256163.s005.tif]
